# A Giant Intrathoracic Malignant Schwannoma Causing Respiratory Failure in a Patient without von Recklinghausen's Disease

**DOI:** 10.1155/2016/2541290

**Published:** 2016-03-28

**Authors:** Epameinondas Angelopoulos, Konstantinos Eleftheriou, Georgios Kyriakopoulos, Kalliopi Athanassiadi, Dimitra Rontogianni, Christina Routsi

**Affiliations:** ^1^First Critical Care Department, School of Medicine, University of Athens and Evangelismos Hospital, Ypsilantou 45-47, 106 76 Athens, Greece; ^2^Department of Pathology, Evangelismos Hospital, Ypsilantou 45-47, 106 76 Athens, Greece; ^3^Unit of Thoracic Surgery, Evangelismos Hospital, Ypsilantou 45-47, 106 76 Athens, Greece

## Abstract

We report an unusual case of a thoracic opacity due to a huge mediastinal malignant schwannoma which compressed the whole left lung and the mediastinum causing respiratory failure in a 73-year-old woman without von Recklinghausen's disease. Although the tumor was resected, the patient failed to wean from mechanical ventilation and died one month later because of multiple organ dysfunction syndrome.

## 1. Introduction

Tumors of neural origin are the most common primary neoplasms of the mediastinum, and most of them are benign [1, 2]. Malignant peripheral nerve sheath tumors in the mediastinum are unusual, accounting for only 0.5% to 7% of mediastinal tumors [3]. In a high proportion these tumors develop in patients with neurofibromatosis, with male predominance. We report an unusual case of a huge mediastinal malignant schwannoma, not associated with von Recklinghausen's disease, which occupied the whole left hemithorax causing respiratory failure in a 73-year-old female.

## 2. Case Presentation

**Figure 1:**
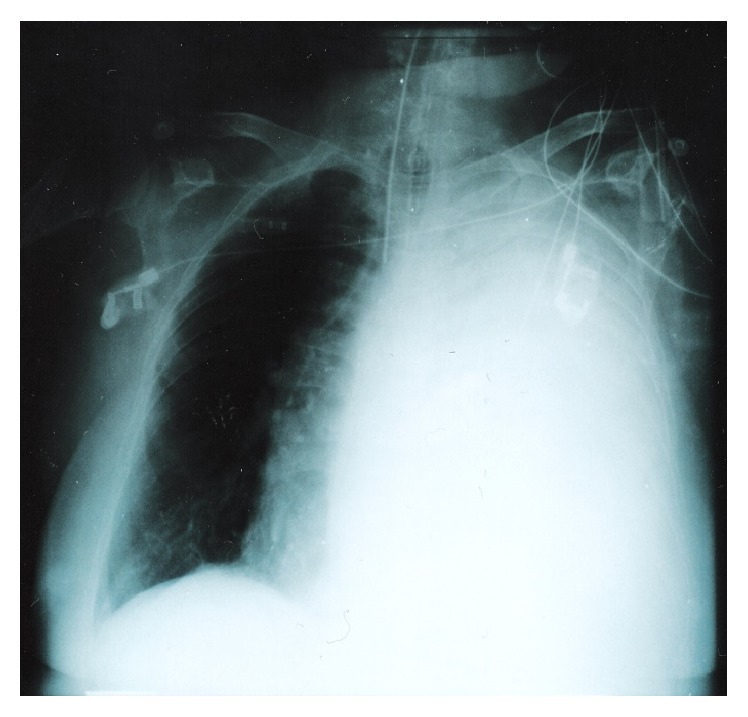
The admission chest X-ray showing complete opacification of the left hemithorax and displacement of the mediastinum to the right.

**Figure 2:**
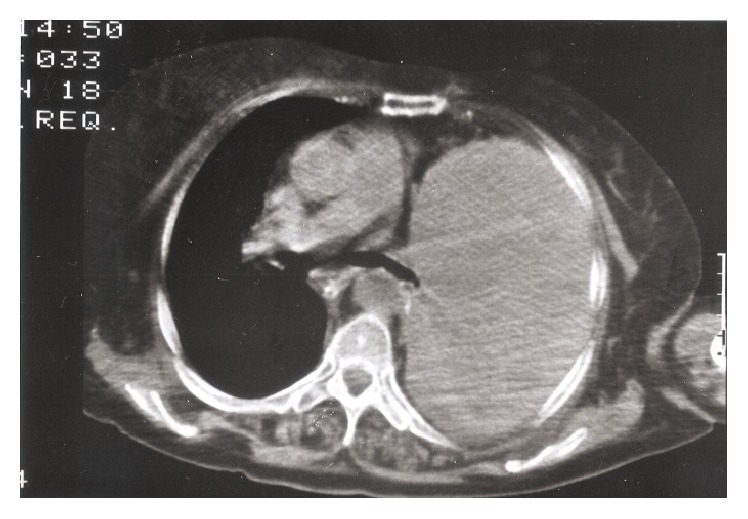
Computed tomography of the thorax showing a large mass in the left hemithorax.

A 73-year-old female was admitted comatose to the emergency department of our hospital with acute-on-chronic respiratory failure. Admission blood gases on 2 L/min nasal oxygen showed PO_2_ 65 mmHg, PCO_2_ 110 mmHg, pH 7.09, and HCO_3_ 32 mmol/L and she was intubated and admitted to the Intensive Care Unit. The admission chest X-ray showed complete opacification of the left thorax with displacement of the mediastinum to the right (Figure 1). A massive pleural effusion was then suspected; thoracocentesis however did not reveal any fluid. A chest CT scan showed a well-defined giant mass of soft tissue density. As a consequence, the mediastinum was deviated to the right, while the left bronchial tree was obstructed because of compression (Figure 2). CT scans of the abdomen and the head were negative. The patient's past medical history was notable for a left pleural effusion of unknown etiology, confirmed by a CT scan, four years previously. She had been hospitalized in a chest disease hospital and the diagnostic work-up had revealed no malignancy. The patient was blind for the last 20 years and had limited physical activity. Dyspnea on exertion and occasional wheezing were reported by the relatives. She had occasionally been treated with inhaled bronchodilators and prednisone.

During the ICU stay, the blood gases on mechanical ventilation were optimized. However, repeated attempts to wean from the ventilator were unsuccessful. On ICU day 17, surgery was decided in order to resect the tumor.

### 2.1. Surgical Findings

**Figure 3:**
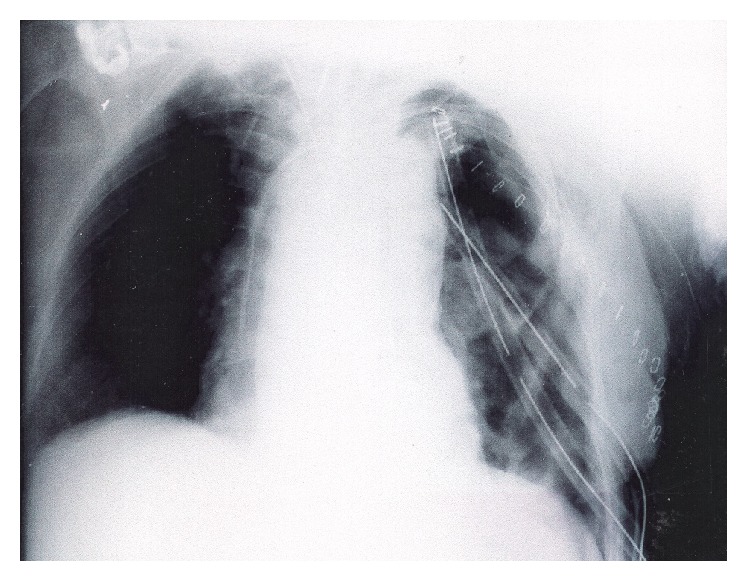
The postoperative chest X-ray showing reexpansion of the left lung.

Left thoracotomy was performed and a giant tumor was found originating from the mediastinum, occupying the whole left thoracic cavity, and compressing the lung to the periphery, not allowing its ventilation. The tumor adhered to both diaphragm and pericardium, compressing them. The resection of the tumor was laborious. Decortication of the left lung followed in an attempt to expand it, and subsequently the patient returned to the ICU. A chest X-ray showed that the left lung had expanded (Figure 3).

### 2.2. Postoperative Course

The first postoperative day was complicated with hemorrhage of the left hemithorax, needing reoperation. During the next days, the patient, although stable, could not be weaned from the mechanical ventilation. Subsequently, she developed sepsis and multiple organ dysfunction syndrome and died on the 30th postoperative day.

### 2.3. Histopathologic Findings

**Figure 4:**
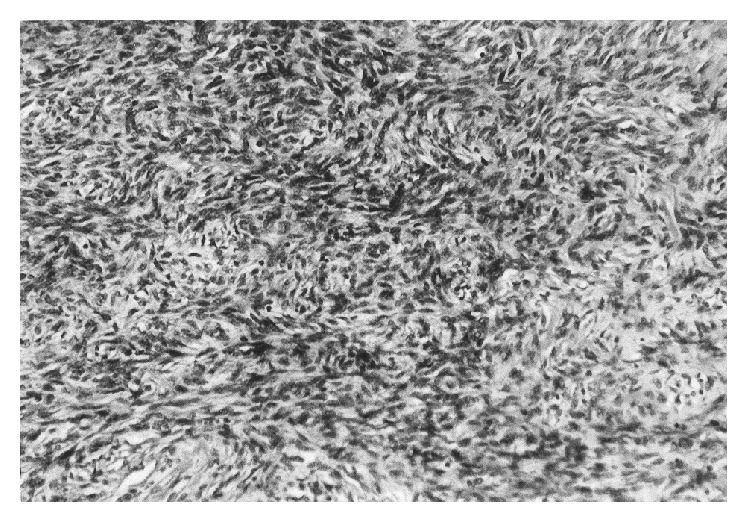
The tumor cells had a wavy appearance; a palisading arrangement was focally observed (H&E ×400).

The main part of the extirpated solid mass measured 35 × 18 × 18 cm. The histological examination of the bioptic material revealed a highly cellular neoplasm composed of pleomorphic spindle cells arranged in solid sheets, short bundles, or interlacing fascicles. The tumor cells had a wavy appearance and a palisading arrangement was focally observed (Figure 4). The neoplasm exhibited numerous mitotic figures and few foci of necrosis. Immunohistochemically, the neoplastic cells expressed only vimentin and were negative to the antibodies of S-100 protein, actin, and desmin, to the myelomonocytic markers lysozyme and CD68 (kP1), and to the endothelial markers. Mostly the morphology and partially the immunophenotype were consistent with malignant peripheral nerve sheath tumor (malignant schwannoma).

## 3. Discussion

Malignant schwannomas account for less than 5% of nerve sheath tumors and approximately half of these are associated with von Recklinghausen's neuromatosis [1, 2]. Intrathoracic malignant nerve sheath tumors are rare. Reviews of collective cases in the earlier literature found that only 3 (1.3%) of 232 solitary malignant schwannomas were located in the mediastinum [4]. On the other hand, only 25 (3.6%) of 688 mediastinal neurogenic tumors were malignant schwannomas [5]. Similarly, only 9 out of 194 thoracic tumors were malignant schwannomas in a later study [6]. There are also a few occasional reports. The tumor presented here occupied the whole left intrathoracic space causing complete atelectasis of the left lung and right deviation of the mediastinum. Such a giant tumor of this type has not been presented and there is not any report of respiratory insufficiency because of the tumor size.

Patients with malignant peripheral nerve sheath tumors are usually symptomatic and present with chest pain, dyspnea, and other symptoms depending on the location of the structures invaded by the neoplasm [3]. The limited physical activity of our patient was probably the reason for her late admission since her symptoms were insidious. Taking into account the fact that in the previous hospitalization only a pleural effusion had been found without a tumor, it may be suggested that the tumor must have grown during the previous 4 years.

The histological and immunophenotyping findings of the tumor were consistent with malignant peripheral nerve sheath tumor (malignant schwannoma). It should be noted that it has been widely accepted that the malignant tumors of the nerve sheath are in their majority negative rather than positive to S-100 protein [7]. Therefore, the negativity of S-100 protein antibody applied to the tumor cells rather supports the diagnosis of malignancy of the tumor of nerve sheath origin.

Although this diagnosis is rarely seen, it must be considered in any unusual thoracic tumor even in the absence of neuromatosis.
